# Navigating the multiple dimensions of the creativity-mental disorder link: a Convergence Mental Health perspective

**DOI:** 10.1007/s44192-023-00051-4

**Published:** 2023-11-16

**Authors:** David G. Angeler, Erin Smith, Michael Berk, Agustín Ibáñez, Harris A. Eyre

**Affiliations:** 1 Department of Aquatic Sciences and Assessment, Swedish University of Agricultural Sciences, 7050, 750 07 Uppsala, Sweden; 2 Neuroscience-Inspired Policy Initiative, Organisation for Economic Co-Operation and Development (OECD) and the PRODEO Institute, Paris, France; 3 Global Brain Health Institute at University of California, San Francisco (UCSF), San Francisco, CA USA; 4 Trinity College Dublin, Dublin, Ireland; 5 Department of Medicine, Stanford Hospital, Stanford, CA USA; 6 IMPACT, the Institute for Mental and Physical Health and Clinical Translation, Deakin University, Geelong, VIC Australia; 7 Department of Psychiatry, University of Melbourne, Melbourne, VIC Australia; 8 Orygen Youth Health, University of Melbourne, Melbourne, VIC Australia; 9 The Florey Institute for Neuroscience and Mental Health, University of Melbourne, Melbourne, VIC Australia; 10 Latin American Brain Health (BrainLat), Universidad Adolfo Ibáñez, Santiago, Chile; 11 Cognitive Neuroscience Center (CNC), Universidad de San Andrés, Buenos Aires, Argentina; 12 National Scientific and Technical Research Council (CONICET), Buenos Aires, Argentina; 13 Department of Psychiatry and Behavioral Sciences, Baylor College of Medicine, Houston, TX USA; 14 The Brain Capital Alliance, San Francisco, CA USA; 15 School of Natural Resources, University of Nebraska-Lincoln, Lincoln, NE USA

**Keywords:** Creativity, Mental disorders, Neurodivergence, Paradox, Strange loop, Convergence Mental Health, Psychiatry, Neuroscience, Complex systems, Brain capital, Transdisciplinary research

## Abstract

**Background:**

This paper discusses a paradox in mental health. It manifests as a relationship between adverse “bad” effects (suffering, clinical costs, loss of productivity) in individuals and populations and advantageous “good” aspects of mental disorders. These beneficial aspects (scientific, artistic and political accomplishments) emanate at the societal level through the frequently unprecedented creativity of people suffering from mental disorders and their relatives. Such gains can contribute to societal innovation and problem-solving. Especially in times of accelerated social-ecological change, approaches are needed that facilitate best-possible mental health care but also recognize creative ideas conducive to beneficial clinical and social-ecological innovations as soon as possible.

**Discussion:**

This paper emphasizes the need to account for creativity as a crucial component in evolving mental health systems and societies. It highlights the need for wide-ranging approaches and discusses how research targeting multiple facets (e.g., brain level, cognitive neuroscience, psychiatry, neurology, socio-cultural, economic and other factors) might further our understanding of the creativity-mental disorder link and its importance for innovating mental health systems and societies.

**Conclusion:**

Our discussion clarifies that considerable research will be needed to obtain a better understanding of how creativity associated with mental disorders may help to create more sustainable societies on a fast-changing planet through innovative ideas. Given the current-state-of-the-art of research and healthcare management, our discussion is currently speculative. However, it provides a basis for how pros and cons might be studied in the future through transdisciplinary research and collaborations across sectors of society.

## Introduction

The new Millennium is commencing with an escalating global mental health crisis with substantial individual, societal and economic consequences. Approximately 450 million people are estimated to suffer from mental health problems, which makes mental health, brain health and neurological disorders one of the leading causes of disability and poor health around the globe [1]. This advancing tide of mental health burden is spot-lit against the receding tide of communicable and many other non-communicable medical disorders. The estimated costs of the consequences of poor mental health are up to 4% of the Gross Domestic Product worldwide [2], and epidemiological modeling suggests that the economic burden of mental illness will rise to $16 trillion by 2030 [3]. Although significant mental health promotion and prevention programs are underway [4], these efforts are likely not enough to manage the loss of human capital [5]. Climate change will likely further aggravate the problem [6] and dwarf the Covid-19 crisis.

The societal implications of mental disorders, which includes a spectrum of diagnoses, etiologies and manifestations related to psychological and brain health factors, are clearly undesirable. However, there is also ample evidence of societal gains due to the creative artistic, philosophical and scientific endeavours and political capabilities of people with mental health conditions and their relatives. This paradoxical relationship between advantageous “good” (creativity and societal innovation) and adverse “bad” (suffering, healthcare costs, productivity loss) aspects of mental health will be discussed in this paper (and referred to as the good/bad paradox). Specifically, the discussion focuses on creativity, often over-represented in people with mental disorders and their families. The paper will examine the creativity-mental disorder relationship from a holistic perspective. That is, it discusses a range of factors ranging the neuronal to social-ecological level that need to be studied for obtaining a mechanistic understanding of the creativity-mental disorder link. We argue that a better understanding of processes may allow to identify circumstances when creative ideas are likely to be conceived and translated into societal innovations and problem solving. We emphasize that substantial research across sectors of society will be needed.

Considering evolutionary biology and spectrally distributed traits and vulnerabilities gives a more nuanced perspective about this good/bad paradox [7–10]. The essay by the evolutionary biologist Theodosius Dobzhansky [11], “Nothing in Biology Makes Sense Except in the Light of Evolution” may be informative here. From an evolutionary perspective, any vulnerability to illness that is persistently adverse to a population should become extinct. However, mental disorders remain highly prevalent and indeed conserved across all societies. The evolutionary explanation is that traits that predispose to illness may also, but do not always, predispose to factors that confer a survival advantage, even if they are a priori not causally related to survival. There are many examples in biology such as sickle cell anaemia. In the case of mental illness, creativity would be a top line exemplar emerging from parallel running advantageous and disadvantageous factors that cannot be easily disentangled. It follows from this evolutionary interpretation that trait and population level factors cannot be separated, and in which both clinical and subclinical groups need to be integrated.

There are far reaching consequences of the good/bad paradox in mental illness. The implications seem counterintuitive, audacious and preposterous: the paradox suggests that approaches are needed to reduce the burden of mental illness, and simultaneously cultivate and foster factors that might also inadvertently lead to adaptive mental health states that are conducive to creative thinking. Examples include states of mild hypomania, soft and reflective “creative melancholia”, and remissions. The relation between external environmental demands, and interoceptive regulatory processes configurates multiple potential behavioral outputs, some adaptive (allostatic load) and other pathological (allostatic overload) [12]. It therefore remains necessary to also manage or mitigate states that are associated with severe impairment and dysfunction, such as full-blown depression or mania in bipolar disorder. Accounting for this tension in mental health, such cultivation may translate the potential of, for example, personality traits inherent in, or associable with both mental illness and creativity into tangible societal benefits.

**Fig. 1:**
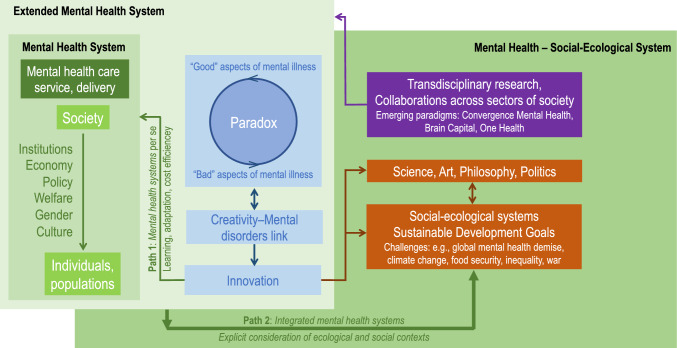
Schematic model demonstrating how “traditional” mental health system can be broadened into “expanded” mental health and combined “mental health—social-ecological systems” model. Path 1 shows innovations of mental health systems for improved (adaptive) healthcare delivery and services that accounts for the pros and cons that arise from the mental disorder-creativity link. Path 2 indicates that extended mental health systems may speculatively facilitate the navigation of complex social-ecological problems through hypothetically spurred creative thinking leading to societal innovations in the sciences, arts and other realms

**Fig. 2:**
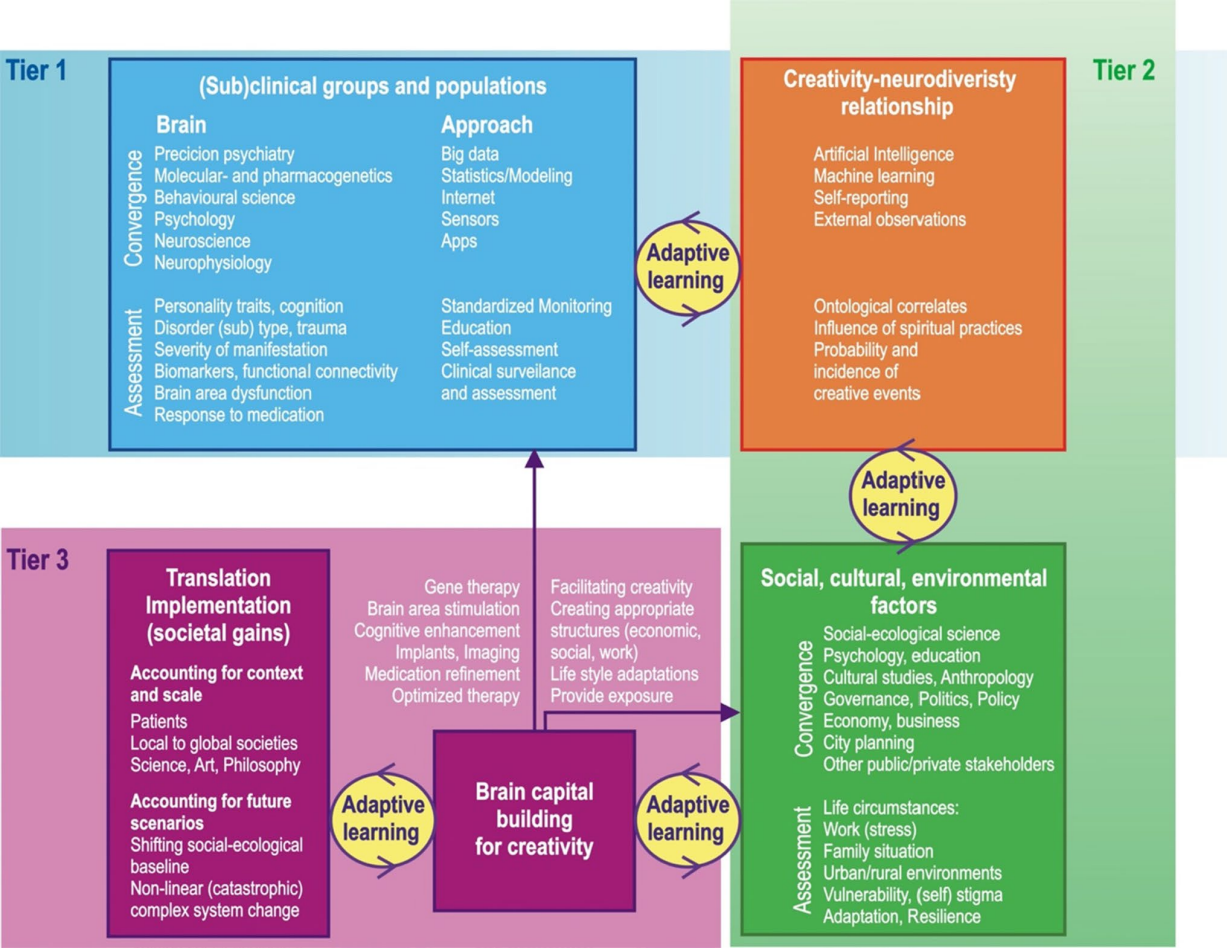
Transdisciplinary research model, based on Convergence Mental Health, for navigating the creativity-mental disorder link. The model consists of 3 tiers: the first and second aim at recognizing brain and environmental/social/cultural correlates with the link, respectively, and the third aims to translate patterns into tangible social benefits emerging from the creativity-mental disorder connection. Every step in the model applies a reiterative transdisciplinary approach for adaptive learning. The model also suggests scenario planning as a tool for envisioning future social-ecological, including mental health, conditions for which a Convergence Mental Health approach, informed by the creativity-mental disorder link, can be specifically tailored. Note: examples are not exhaustive and serve for demonstration

We present a theoretical discussion that examines the good/bad paradox, informed by the creativity-mental disorder link (Fig. 1). Creativity, which despite difficulties with operational definitions [13], is an important tenet of discovery and innovation [14] and often over-represented in people with mental disorders. We discuss that a better mechanistic and holistic understanding is likely feasible when (1) the circumstances (neurological aspects, personality traits, social-ecological environments) leading to “Eureka moments” are studied; and (2) pathways are created that facilitate and optimize the translation of ideas into tangible societal benefits. Although such an improved understanding may be principally possible, we also highlight that substantial transdisciplinary research will be required [15] (Fig. 2). However, we are confident in our speculation that ultimately novel opportunities may emerge to confront and potentially solve vexing social-ecological challenges, such as climate change on a rapidly changing planet Earth [6, 16, 17]. Our discussion pinpoints the importance of mental health care users to contribute innovative ideas and solutions to many of these problems. This notion clearly resonates with the increased imperative for consumer co-design in research and partnership in clinical mental health service delivery [18].

Before discussing our approach, it is critical to consider relevant terminology about what constitutes mental illness in relation to creativity.

## Creativity-mental disorder links: terminology

Many terms such as psychopathology or mental illness are laden with potentially negative value judgements, yet such terms have been commonly used in the literature about creativity-mental disorder relationships. Many people may not think of themselves as mentally ill but may experience a co-occurring mental health condition, such as depression. We distinguish the idea of neurodiversity, which is a non-normative condition of all humans [19], from neurodivergence, a term used to apply to individuals or groups who are distinct from an assumed cognitive/neurological (neurotypical) norm but which comprise variants of neurodivergence [20]. The deviations from such norms can manifest in a range of neurodivergent types, including autism, dyslexia, attention deficit disorder and chronic mental health disorders such as bipolar disorder, schizophrenia, obsessive–compulsive disorder, anxiety, and depression.

Some authors recently referred to neurominorities as an umbrella term for the subset of neurodivergent conditions [21, 22]. Such a conceptualization is useful because some neurodivergent people may exhibit mental health problems while others, crucially, do not. Such distinctions may also facilitate to disentangle the ‘dysfunctional’ aspect from the ‘neurological difference’ aspect associated with neurodiversity while accounting for whether or not neurodivergent people have mental health problems and whether or not they flourish and have a good quality of life [23]. In this paper, we will use mental disorder in the context of neurominorities to emphasize creativity at the level of individuals and populations with mental health conditions, acknowledging that creativity, a term which is itself difficult to operationalize, can vary markedly among the whole spectrum of neurodiverse individuals.

## Toward a transdisciplinary approach

We suggest a model which discusses the relevance of studying the creativity-mental disorder link in the context of the good/bad paradox from two viewpoints (Fig. 1). First, we suggest that mental health systems per se might benefit from the creativity-mental disorder link. That is, mental health care users may contribute novel ideas that may innovate clinical mental health service delivery and spur research (Path 1 in Fig. 1). Recently, a creative user with bipolar disorder converted iMoodJournal monitoring data into music, which provides a potential innovative clinical approach to facilitate coping with patients’ mental health conditions and better insight into the self [18]. This study provides an example that creativity-driven patient co-production has the potential to innovate and enhance the management of mental health of entire populations, likely leading to reduced cost and improved therapy outcomes. If such novel approaches are widely applied at the mental health system level, mental health systems may become dynamic, adaptive and “self-innovate” through health care service and delivery approaches emerging from an iterative learning process that may be creativity driven. Accounting for creativity, contextualized within a system of human affect and emotions [24], may complement and inform current mental health systems targeting a plethora of factors such as, for instance, public health and learning [25], patient co-production and participation [26], gender issues [27], race and poverty [28], environmental disaster response [29] and adaptive and transformative approaches to health care systems and management [30]. Such a process may lead to an extended mental health system in which the creativity-mental disorder link becomes a crucial component (Fig. 1).

Second, creativity-driven learning and improvement processes in mental health care systems will ultimately benefit entire societies and facilitate the navigation of complex social-ecological problems, likely contributing to problem solving (Path 2 in Fig. 1). Mental health systems are components of, embedded in, and critically influenced by broader social-ecological systems dynamics [30]. A combined “Mental-Health—Social-Ecological System” view may provide the necessary frame for studying the creativity-mental disorder link holistically from a societal perspective; for instance, by accounting for environmental and social factors influencing the dynamics of such systems. Collaborations across sectors of society may result in a learning process, partly influenced by innovations in the sciences and arts through people with mental disorders, in which the dynamics of such a broader “Mental-Health—Social-Ecological System” may be studied reiteratively.

Given the need for creativity to confront such complexity, in the next section we will examine the creativity-mental disorders link. This examination will account for creativity and divergent thinking [31], which can be often associated with mental health [32]. Our theoretical model will show the overwhelming complexity and challenges that science and health care face for balancing “the bad” and “the good” aspects of mental illness. Our model is therefore no prescription but meant to highlight the need for an “eclectic approach” [13] to capture creativity. The model suggests how such an approach can be operationalized and implemented using transdisciplinary research [33]. It concludes with highlighting the importance of dissemination to increase awareness, potentially reducing stigma, and accounting for ethical issues.

## Mental health and creativity

It is acknowledged upfront that it is not our aim to review the complexity and tensions surrounding creativity and mental health that has been extensively discussed in the literature. We rather use creativity as a broad umbrella for contextualizing and operationalizing our model and spur research about creativity and mental disorders using transdisciplinary research. We also do not discern between situational (building existing knowledge) and fundamental (generating genuinely novel knowledge) forms of creativity [34, 35].

Creativity is not limited to the broad spectrum of mental health conditions [13], for example in creating coping mechanisms for alleviating adverse conditions [36]. Creativity is clearly present in daily life, such as performing at work, dealing with interpersonal issues, managing painful emotions, or cooking dinner [37]. Groundbreaking ideas relevant for societal innovations may eventually emerge through such daily endeavors, for instance through discussions at work. Such processes are mediated by synergies between language, interoception, sensorimotor activity, memory, social cognition, and decision making, to name a few [38]. Creativity and innovation are frequently due to the efforts of individuals and groups and are often particularly high when representatives from different sectors of society (academia, public, private stakeholders) and areas of expertise engage in multidisciplinary forms of inquiry [39]. However, shared factors for mental ill health may benefit such multidisciplinary collaborations, potentially facilitated by the ability of the mentally ill to think divergently [32]. Building on this ability to capitalize on and translate creative divergent thinking into tangible products, the Swedish IT consulting company (UNICUS) considers Asperger syndrome as a “superpower” and thus an employment criterion [40]. Different elements of neurodiversity may have adaptive benefits in different domains for example Aspergers and mathematics or computing.

With specific regard to creativity and its link to mental disorders there are currently two perceptions in the neurological, psychological and psychiatric sciences. One reflects some research showing no or spurious links between creativity and mental disorders [41–43]. This is due to studies often suffering from methodological issues (design, sample size, statistical power, metrics with poor psychometric performance). It is also recognized that the detection of such a link can be difficult in clinical and scientific studies. Mental health symptoms and their manifestations can be highly variable and transient across individuals and populations (clinical and subclinical groups). Factors driving such diverse phenotypes as creativity and mental health do not operate alone and interact with other biological and environmental variables. There may also be dose effects in play such that a small quantum might be advantageous and larger quanta disadvantageous. This can similarly be explained with phenotypic variation reflecting genetic variability, often unique symptom profiles and environmental factors mediating these [44]. Mental disorders therefore qualify as gradients within spectra rather than delineated categories. This high variability makes it difficult to unambiguously pinpoint the mechanisms of the creativity-mental disorder link within established categories (i.e., diagnosed type of mental disorders [e.g., bipolar], variability within disorders [rapid cycling, Type 1 and Type 2 bipolar], and comorbidities [e.g., substance abuse disorder, anxiety disorder, and/or ADHD co-occurring with bipolar]).

Closely related is the variability of creativity itself which manifests at a scale of gradations that ranges “mini-c”, “little-c” to “Big-C” creativity [45]. Even within these magnitudes of creativity, the variation is huge [46]. It follows that the severity of symptom expression during episodes can interfere with creativity [47, 48] and patients’ personalities (e.g., openness rather than schizotypy [49]) and social and cultural aspects may further complicate the picture [50]. Consequently, a range of sources of variability can introduce “noise” and swamp the detection of mental disorder-creativity links which have been amply documented in the literature [51]. It’s also true that creativity often requires a flash of creative insight followed up by much plodding sequential development. Such flashes can occur for example in a period of hypomania or mania and are developed long after the episode has settled.

Notwithstanding the current tension around links between creativity and mental disorders, our interest is to provide a brief overview of links documented in the literature to inform our model. The chosen examples are non-exhaustive and disparate. They only provide a glimpse of the enormous variability and diversity in terms of the manifestations of such a link in populations with different occupations, age, mental disorders, and clinical subgroups or through interpersonal relationships. The rationale is to suggest that this diversity needs to be taken into account for driving future basic and clinical research on this topic [50], and devising holistic science and mental health systems.

The relevance of the creativity-mental health link pertaining to different professions is well documented [52]. For instance, comparing different occupations, Simonton [46] found positive correlations between “psychopathology” and creativity for eminent writers and artists, and nonlinear associations for scientists, composers, and thinkers, with the degree of mental ill health differing within the latter group. This result supports that psychopathology varies with the domain of creative achievement. Associations with mental illness are less prominent in scientific creativity than artistic creativity, although variability exists, again, if different scientific fields (nature *vs* social sciences) and art styles (poetry *vs* non-fiction writing) are considered. The creativity-mental disorder link is less pronounced in scientists because of the structured scientific process constraining flexibility in the translation of ideas. However, scientists with mental health conditions achieving eminence have adopted research approaches that often revolutionized entire disciplines. In contrast, their healthy counterparts were more likely to achieve eminence once paradigms became established [53]. Disregarding the relevance of eminence for contemporary research, this pattern has important implications for the current global environmental sustainability crisis. It is increasingly recognized that current siloed research approaches based on existing paradigms are unlikely successful to confront the high complexity inherent in this crisis. Such complexity may only be dealt with by revolutionary approaches emanating from novel paradigms [54].

That representatives of creative professions (writers, visual artists) and their family members suffer a higher-than-average prevalence of mental ill health was also found by Kyaga et al. [55]. Their study showed that this link was evident in certain groups of patients with proclivity to or diagnoses of bipolar disorder and healthy family members of schizophrenia patients but not in individuals with unipolar depression. Other studies also found higher risk of affective disorders, alcoholism and drug use but not necessarily schizophrenia in eminent people of literature and the arts [56, 57]. This indicates the need to account for context dependencies of mental disorder-creativity links in research. The above-mentioned evolutionary perspective that emphasizes the need to account for individual and population factors (clinical and subclinical individuals in families) may inform such contexts. The consideration of ontogenetic factors may also be needed because many creative individuals achieving eminence in their disciplines (e.g., Kurt Gödel, Vincent Van Gogh) showed a decline in productivity when experiencing severe mental illness [51].

Creative variability can also be pronounced already in early stages of life, and creative manifestations may occur in both clinical and subclinical groups of children and adolescents. For instance, creativity was found to be higher in children and adolescents with attention deficit hyperactivity disorder (ADHD) who display clinically moderate levels of dysfunction [47, 58]. This was also found in subclinical (healthy) children with a high degree of either psychoticism or schizotypal traits relative to their low trait counterparts [59, 60]. One ramification of this pattern is that psychosocial environments need to be created to support those with subclinical conditions. Care needs to be taken to minimize risk for healthy groups with a proclivity to eventually trigger mental health conditions from tipping over into a clinical spectrum that requires substantial health care. The implication, especially for the creation of well-functioning mental health systems is that proactive, adaptive management is more cost-effective than reactive approaches that often require substantial health care intervention, which has been discussed, for instance, in the context of bipolar disorder [61]. Furthermore, from a scientific perspective, subclinical groups allow to study creativity-mental disorder links without confounding factors (e.g., psychopharmacological treatment) [51].

Despite the disparity of the patterns described above, the creativity-mental disorder link seems to have underlying commonalities that emanate from genetic [62], neural [63] and psychological [64] mechanisms, driven by genetic evolution [65, 66]. These commonalities include cognitive disinhibition, whereby more stimuli permeate conscious awareness that facilitates increased conceptual and unconventional connections and thinking, adoption and entertainment of novel and alternative viewpoints, and a high tolerance for ambiguity [51, 67].

In summary, the creativity-mental disorder link operates at the intersection of, and is contingent on, a plethora of factors including neuronal, psychological, cognitive, brain structural, and neurophysiological features mediated by evolutionary, genetic and personality factors, and social and cultural environments [50, 65, 67]. All these factors are relevant for understanding mental health systems as complex and multidimensional ecosystems [30]. In the next section we discuss this complexity. The focus is on Convergence Mental Health [68], an emerging paradigm that builds on transdisciplinary collaborations between medical, social, statistical, big data, artificial intelligence and environmental scientists, engineers, artists, educators, statistical modelers, economists, clinicians, people from spiritual realms and other stakeholders (e.g., decision makers). Convergence Mental Health therefore helps operationalizing transdisciplinary research approaches tailored for what we consider extended mental health systems and mental-health—social-ecological systems (Fig. 1). This makes Convergence Mental Health as a form of transdisciplinary collaborations well suited in two ways: (1) It targets the development of preparedness and coping/resilience mechanisms to improve mental health and establish treatments for psychopathologies and neurodegenerative diseases (dementia, Alzheimer) [69]. The creation and promotion of “resilient brains” may find application to foster creativity in relation to latent vulnerability and may ultimately have potential to reduce mental illness through mental ill health, a paradox in itself. (2) Due to its broad holistic focus, Convergence Mental Health integrates research and transdisciplinary collaborations for creating social-ecological resilience and sustainability on a rapidly changing planet. Convergence Mental Health by definition is open for collaborations with other interdisciplinary platforms in social-ecological research such as One Health [30].

## Navigating the model

The applications of a Convergence Mental Health approach for navigating the model and potentially boost creativity linked to mental disorders are vast and likely inexhaustible. We can therefor only provide a teaser of such applications at this stage. Our discussion is on purpose speculative, vague and partly not evidence-based. This is on one hand due to the novelty of Convergence Mental Health and on the other hand knowledge of individual research domains in mental health and other (social-ecological) systems not having been studied in synergy yet. Predictable outcomes, especially pertaining to the creativity-mental disorders link, are therefore difficult to envision, partly because of fast technological progress and societal change creating uncertainty. Our discussion of approaches (Fig. 2) is therefore theoretical at this stage.

Forgeard and Elstein [37] highlighted that creative thinking constitutes an important yet understudied process, but which ultimately can be defined, operationalized, assessed, and (if adaptive) refined and improved. We concur with these authors. We borrow from the emerging Convergence Mental Health literature [33], which has shown promise to combat mental illness by unifying research from individual (sub)disciplines into an overarching approach useful for navigating the good/bad paradox. Our model (Fig. 2) lets the creativity-mental disorder connection and its implication for mental health systems and societal gain take a central role. Therefore, the model considers that current Convergence Mental Health approaches are useful for abating the effects of mental illness. At a societal level they can be tweaked towards assessing, operationalizing, translating and implementing positive (creative) aspects of mental disorders and those with shared operative drivers while minimizing its negative effects including costs for mental health systems.

### The model

Our model is based on a three-tier, multi-scalar approach (Fig. 2), wherein every tier may be conducive to transdisciplinary research on its own. Tier 1 and Tier 2 serve to recognize patterns of creativity-mental disorder links pertaining to populations and socio-cultural factors, respectively. Tier 3 considers translating these into tangible societal gains. Rather than comprising isolated components, these tiers inform and are informed by the other tiers in the model. The following description of model tiers will examine these points with more detail and highlight the need for a better integration of neuroscience, psychiatry, technology and a range of social factors in mental health systems:

#### Tier 1

This tier considers the identification of patterns and underlying mechanisms of creativity-mental disorder links in clinical and subclinical populations. It targets the interplay of brain-level factors, such as genetic, molecular and cellular functioning, and cognitive, behavioral and human body systems as building blocks of creativity. This tier also suggests that the analysis can be extended to (subclinical) groups and populations in different realms of inquiry, such as the arts, sciences and philosophy at different spatial extents. These considerations may help to provide a sound scientific basis about commonalities and differences in the creative process within and across populations from an evolutionary perspective.

New technologies (Apps, internet, mobile phones, artificial intelligence) allow monitoring of external (situations, social interactions, environments) and internal (mood, physiology, “digital phenotypes” and ultimately brain signatures) conditions across time and space. Analysis of social media data (e.g., Facebook, Instagram) may provide complementary insight into mood states that might be conducive to or inhibit creative moments based on color, meta data and algorithmic face recognition [70]. These technologies can thus be helpful to identify what helps or hinders moments of “flow state” which are key for creativity.

The use of wearable smart electronic devices may add support through real-time monitoring of biometric data on autonomous nervous systems activity, sleep quantity and quality, voice analytics, and social and physical activity [71]. Including brain-level data (neuroimaging, molecular/inflammatory biomarkers, functional connectivity in neural networks, dysfunction within and across brain areas) and electronic medical records could potentially add further useful information. Signals can be analyzed through artificial intelligence and machine learning to reveal the probability of occurrence of mood states in clinical groups that are most conducive to idea generation, such as states of remission and potentially mild hypomania or soft creative melancholia. A nominal level of function is probably crucial for developing original ideas rather than fleeting thoughts. Similarly, thresholds may be identified upon which symptomatology may become too severe and creative thinking impaired, such as, for instance, in severe depression and psychosis. Early recognition based on real-time monitoring using the technological approaches discussed above may be used for delivering psychotherapy (e.g., through chatbots, pet robots or potentially virtual reality [71]) to prevent and mitigate severe episodes and provide the right level of care at the right time.

It follows that Tier 1 assessments needs to integrate multiple disciplines from the basic and clinical sciences, as well as areas related to the delivery of therapies. Convergence of insight research, cognitive science, neurostructural and neurophysiology research, technology, robotics, statistics, psychology, molecular biology, pharmacogenetics, pharmacokinetics, and precision psychiatry exemplify the magnitude of this effort.

#### Tier 2

This tier focuses on social, cultural, demographic, economic and ecological correlates of the creativity-mental disorder link in populations. An “ecosystem view” of mental health systems is useful for a convergence approach at this level [72], because the complex, adaptive, direct and indirect relationships between factors operating at the brain, cognitive, behavioral (internal factors), and environmental and socio-cultural level (external factors) can be considered. The interactions of these factors ultimately shape the feedbacks, stability and dynamics of a mental health ecosystem [72]. Given the latitude and complexity at this scale, this tier speculates broadly how the cross-pollination of the following fields and others may facilitate understanding of the life circumstances of people outside the “bounds of normality” and how these circumstances may influence their creativity potential: social-ecological, cultural and demographic sciences, gender and anthropology research, economy and equality studies, city and landscape planning, (cognitive) psychology and education, governance, among others.

Life conditions such as work, family and other social situations, access to resources, freedom, basic incomes and needs, access to nature and green city infrastructure, physical blight in urban environments have been abundantly documented in the literature to affect vulnerabilities to stress, adaptation and coping potential and creativity and productivity. Ultimately, life circumstances mediate mental health awareness, behavior, resilience and response ability as a function of their experience, education and personality traits [73] (Tier 1). Tier 2 envisions that generating massive amounts of social, ecological, economic, cognitive and other data and subjecting them to increasingly powerful artificial intelligence and deep learning tools may help to illuminate how the entire mental health ecosystem from individuals to social factors mediates creativity. Insight research, which studies spontaneous creative manifestation, may provide a useful basis for such explorations. However, such research has so far been problematic because of the lack of agreement among psychologists of its definition and ambiguity. This is the result of the phenomenological nature of insight, and the difficulty in catalysing its occurrence and experimental induction [74]. Notwithstanding, as more data accumulate, reiteratively assessing these associations may provide a refined picture about the probabilities and incidences of creative events manifesting and complement the picture provided by Tier 1 (Fig. 2). Such adaptive “narrowing down” of probabilities and incidences will likely not fully resolve creativity’s “shyness” (its serendipitous, spontaneous and unanticipated manifestation), but the combination of Tier 1 and Tier 2 may reveal patterns of when and under which circumstance creative events are more likely to happen. The resulting information may have many practical applications, for example in redesigning education systems including those in mental health system [33].

Once patterns are generated, environments may be tailored to optimize potential creativity in (sub) clinical groups. Creative spaces may be needed that require the alleviation of stressful factors that propagate through society, a seemingly impossible task. However, hypothetically, the creation of such spaces can borrow from, and be inspired by discussions already taking place in academia about structural changes in policy across public and private stakeholder sectors and funding schemes [33]. These discussions could inform models for fostering creative environments for the mentally vulnerable and ill while providing them with the necessary stability to sustain their livelihoods and mental well-being through redesigning mental health and occupational systems. How such environments could look like in the future remains to be seen but may be informed by people with mental disorders conceptualizing and creating social and environmental factors conducive to their mental ill health and mitigate these.

The creation of creative spaces will clearly need the incorporation of a policy dimension that considers the wider spectrum of mental disorders as a potential context dependent strength rather than a limitation. “Broken minds” will need tailor-made dynamic, adaptive environments for sustaining livelihoods and optimal functioning to make creativity potential fully blossom. Accounting for “orchid” (i.e., sensitive, fragile, susceptible) and “dandelion” (flourishing and surviving under most circumstances) type of personalities may be useful [75]. The creative potential will likely be suppressed through rigid, static, one-size-fits-all “broken bone” health care and social security approaches. Such considerations are crucial for mental health system building and the translation of creative ideas into practice. This aspect will be further discussed in the next tier.

#### Tier 3

This tier considers the building of refined and extended mental and occupational health systems as a means to optimize the creative potential associated with mental health and ultimately translate it into societal gains. This can focus on mitigating mental disorders to foster liberal thinking abilities and the capacity for making novel connections between existing paradigms [31, 67, 76]. Such modulation may target, for instance, five mutually non-exclusive approaches rooted in the cognitive sciences [77]: (1) Minimizing the mind’s conditioning and being mindful of such biases by consciously keeping an open mind to reduce the probability of conditioned behavioral responses; (2) Increasing the odds of manifesting a low-probability creative idea by persistently examining an unsolved problem of interest; (3) Exposure to unlearned stimuli that are a priori ambiguous, such as abstract paintings or Zen koans, to facilitate Eureka moments. Even reported symptoms themselves may facilitate such moments; for instance, when delusions and hallucinations in schizophrenia inspire solipsism [78] and transcendental idealism informed by quantum mechanics (DGA, unpublished); (4) Facilitation of unconscious processing of information and stimuli; and (5) Enhancing the probability of creative events by working and talking with other people, especially from diverse backgrounds. Inherent in these points are the creation and enhancement of adaptive prospection through psychological flexibility; that is, the ability to effectively adapt one’s cognitions, emotions, and behaviors to a situation at hand [79, 80]. Also, other potential mechanisms benefiting creative activities, including adaptive emotion regulation, flow, meaning-making, or growth from adversity could be targeted [37].

Possibilities for research are potentially vast, ranging disparate approaches from precision psychiatry to mindfulness. For instance, electroencephalogram studies and functional magnetic resonance imaging have found that problem solving requiring insight involves different areas of the brain [81]. Research may focus on if and how such patterns might influence Eureka moments. Other approaches such as spiritual practices (yoga, meditation), which can enhance flexibility on both executive and metacognitive levels may also be useful [82, 83] (but see [84]).

Efforts will require a broad transdisciplinary collaboration to ultimately identify and recognize creativity and divergent thinking potential. These collaborations will also need to be complemented with communication for conveying creative artistic and scientific findings to the broader public and stakeholders. Successful communication will be important for ultimately translating creative ideas into societal innovation and gains in the long term. These points will be further elaborated in the next steps:

#### Identification and recognition

Identifying and recognizing creative ideas is potentially extremely challenging, which can be exemplified with the following, non-exhaustive examples. The outcome of creative processes may result from small c to big C ideas. However, the perceptions of what or how good a creative idea is can be highly subjective, especially in the arts, and not easily recognized. This subjectivity may lead to entirely missing or recognizing late the potentials for societal gains, making the implementations of ideas highly uncertain. Complexity is likely added by societal factors such as the scientific mainstream being skeptical to new perceived radical ideas that break with existing paradigms [85]. Stigma towards the mentally ill risks the rejection of creative ideas, but outreach may change this problem (see below). Also, at the individual level, reporting of recognized Eureka moments may be inconsistent, causing incongruence between documented mood states influenced by environmental factors and perceived creativity, obscuring patterns.

To deal with such challenges transdisciplinary collaborations may build on work between educators, social groups, including family and friends, close to creative consumers and careers, technologists, internet specialists, analysts and web designers. Publicly open online data platforms can be designed where people may upload documentation of their creative outcomes, be it visual art work, poetry, music, philosophical thought or scientific texts. Many emerging platforms targeting these different areas of science and the arts have already found a foothold in social media (Facebook, Instagram). A platform that integrates such structures may accumulate massive data that become conducive to scalable pattern recognition, machine learning and artificial intelligence analyses to identify potentially deviant patterns inherent in existing art and science paradigms. That artificial intelligence can be a powerful and useful tool for such an approach is already manifested in machine learning creating art and even art galleries themselves [86]. With increasing computer power, advances in quantum computing and algorithm performance pattern recognition may further be facilitated by automatically comparing the information in such a platform with the disparate information present in the broader internet of things (e.g., the peer-reviewed scientific literature). Such an approach may borrow from techniques already used in biological image analyses [87], stock market forecasting [88] or cancer diagnosis [89].

#### Dissemination and uptake

This task has the main aim of creating awareness of novel artistic and scientific ideas and their implementation across different spheres of society. For instance, although it is a fundamental human ability to identify and value novelty, the broader public and decision makers do not have the necessary training for valuing novel ideas in very specialized areas such as the sciences and philosophy. Therefore, dissemination and uptake will likely vary between the arts and sciences. While ideas may be made accessible for the science community through peer reviewed outlets, uptake by the public and policy makers will need substantially different approaches. Collaborations between the arts and sciences have recently been used to express complex scientific issues artistically; for example, through the translation of data about political crises, environmental degradation, and mental illness into music [18, 90, 91]. The rational for such an approach is to appeal to peoples’ emotions (through the arts) in addition to their logic (through the sciences), likely facilitating increased learning, awareness and engagement [92].

These examples show that interdisciplinary approaches may facilitate the communication and dissemination of creative findings. Ideas can be conveyed through interactive venues and multiple channels, including journalism, through popular science communication, social media, films, museums, art galleries and conferences, blogs and other platforms. Dissemination can also emphasize the positive, creative aspect of mental illness, potentially reducing stigma, capitalizing on the myth that great ideas can be born in the mind of a “mad genius”. The movie “A beautiful mind” which celebrates the schizophrenic Nobel laureate John Forbes Nash serves an example. It also communicates the enormous struggles of Nash when acute illness inhibited his functioning and creativity. Similarly, the story of Vincent Van Gogh shows that he was unable to paint when severely ill, and even when in Hospital Saint-Paul in Arles, only began to paint when his illness began to subside. Broad dissemination and exposure of ideas to the public may thus be useful because emerging views from the public might feed back to the ideas originally conceived by patients which holds potential to refine these ideas. Public engagement has the added benefit to democratically inform decision makers about designing policy that can turn creative ideas into practice supported by the public. Translating science will require active decision-making to bridge the steps between idea conception, feasibility and rigorous evidence-based proof of concept—a process which can last decades [93]. However, implementation could potentially be accelerated through a variety of measures, including e.g., specifying implementation strategies for different target groups (from individual to groups to varying subcultures), assessing the feasibility and cost-effectiveness of these strategies, and estimating the amount of refinement of organizational and system processes involved in implementation [94, 95]. Such a complex process may likely not be needed for translating art, as new paradigms may become more easily assimilated into society.

#### Scenario planning

The model emphasizes adaptive learning to successively refine our current understanding of creativity-mental disorders links within and across tiers, thereby obtaining a holistic systems view of creativity and mental disorders from individuals to societies. It also suggests that scenario planning, an approach widely used in social-ecological research [96], may be useful for envisioning different potential future social, ecological and economic realities, which may help for adapting or transforming mental health systems. Scenario planning may be particularly useful to account for projected dystopic futures globally affecting brain health. Large-scale conflicts and war, inequity, climate disasters, refugee migration, food insecurity and lost productivity because of communicable to non-communicable health diseases will burden people’s mental health [97–99]. Catastrophic and irreversible change in climate [100] may exacerbate these problems and affect low-income and high-income countries differently at local, regional and continental scales.

Accounting for such different scenarios may provide opportunities to actively envision and provide mental exposure to future social-ecological problems including economic costs for mental health systems. The broadness and complexity of such problems requires creative solutions. Mental illness often correlates with broad and synthetic thinking [67] and results from preparedness, desire and opportunity [101]. It is likely possible that, based on the above-mentioned considerations [77], this envisioning/exposure increases the probability of useful creative solutions to be conceived. It needs to be seen, however, whether this speculation may turn into fact should specific scenarios become real in the future.

## Conclusion and outlook

Mental disorders are unlikely to be eradicated any time soon, if ever, and societal and individual costs will be pervasive. There is a need for trading off “the good” and “the bad” in mental disorders, which we have conceptualized as a paradox. Navigating this paradox for creating well-functioning, expanded mental-health social-ecological systems will require an unprecedented transdisciplinary approach for finding tradeoffs between the societally beneficial aspects (creativity) and costs which mental problems incur. The creation of such complex mental health systems requires the public, mental health care professionals, politicians, scientists across realms of inquiry (e.g., ecology, social and health sciences) and other stakeholders to interact in creative, democratic dialogues. Most importantly the engagement of people with lived experience of mental illness and their loved ones will be crucial for building mental health systems [26].

The question may be raised whether it is worth the effort changing mental health systems to account for creativity of likely a few individuals within large populations of people. The answer is yes because history has shown that the creativity of a single person with a mental health condition can lead to new paradigms that gain a foothold in current societies. Bipolar Ludwig Boltzmann pioneering statistical mechanics or schizophrenic John Forbes Nash proposing game theoretic approaches that play a central role in current economics are but a few examples. Major efforts are already underway to improve mental health delivery and services to facilitate mental well-being and functioning. The paradox inherent in the link between creativity and mental disorders considered here must therefore not be understood as an independent component for study. It should rather be seen as an integration in current efforts to improve mental health systems. Accounting for the creativity-mental disorder link in this endeavour is not only of scientific but also clinical interest.

We conclude by highlighting that our model is theoretical and a simplification of the complexity that will surround the implementation of transdisciplinary research. Because of its range and magnitude, proofing the model empirically is currently elusive. It may also be considered as overly speculative and bordering science fiction although with shards of validation to date. Notwithstanding, there is a long history of scientific breakthroughs having converted widely rejected, provocative speculation into tangible benefits. We therefore regard our model as an openminded attempt to be inclusive rather than exclusive. We also consider time as the best judge of its utility, particularly for the creation of extended mental health systems and mental-health social-ecological systems envisioned in Fig. 1. Our three-tier approach may serve as a cornerstone and inspire how to deal with the different levels of complexity operating within and across tiers.

Several factors (application, privacy and ethical issues) may challenge the implementation of such an approach. The following non-exhaustive examples draw from Eyre et al. [33]:

Regarding application, starting with too much complexity, Convergence Mental Health may be susceptible to obscure creativity-mental disorder relationships. The art of such an approach will be to identify the proverbial “make things as simple as possible but not simpler” (Albert Einstein); that is, identifying crucial factors and scales, and once identified, adaptively improving knowledge about mental health-creativity links. This will need to account for the pros and cons of current technology. This can be exemplified with, for example, neuroimaging at the brain level. Monitoring at scales and frequencies necessary for revealing subtle structural and neuronal brain states that may lead to creative moments is currently unfeasible. Also, despite the power of artificial intelligence and accumulation of big data, creative and accurate solutions will still need human experience and knowhow. For this, not only specialist knowledge but also literacy outside the expertise of participants in transdisciplinary collaborations will be needed. However, structures within and outside academia are still entrenched in siloed approaches for professional training with latitude. So are funding schemes for research and practice, although there are initiatives emerging that support transdisciplinary approaches [33].

Cultural and socioeconomic factors need also consideration. Finding sufficiently large numbers of participating individuals representing social, cultural, economic diversity may be challenging; partly because of potentially negatively biased perceptions of patients and social stigma in some cultures and societal sectors. Vulnerable, marginalized people and countries with substantial economic hardship may face difficulties to find the necessary resources for implementing a sound transdisciplinary approach. Access to internet, smartphones, healthcare and computer literacy in poor communities and age cohorts are examples that may challenge transdisciplinary research.

There remains a need to account for harmful injustice and violations of human rights in health care, specifically among people with mental and physical disabilities [23]. This spans an unduly dismissal of testimony because of prejudiced beliefs regarding minority groups; a community’s shared vocabularies being structured in a way that unfairly distorts or stifles understanding minority groups; the operation of negative stereotypes pertaining to race and gender, for instance, when a person’s testimony is doubted, dismissed or given low credibility due to sexist or racist prejudices on the part of the listener. It will be crucial to make all voices heard and use interpretive tools available to people with mental disorders to facilitate them providing their testimonies and interpretations.

In summary, there is a clear need to account for potential far-reaching ethical and privacy issues that need to be accounted for in policy making [102]. “Responsible innovation” [103], which has the goal to safeguard personal brain data and other information and anticipate and monitor potential unintended use/misuse paves the way for addressing such critical issues.

## Data Availability

Not applicable.
